# Changes in Material Hardship During the First Year of the COVID-19 Pandemic

**DOI:** 10.1001/jamahealthforum.2021.5213

**Published:** 2022-02-25

**Authors:** Brendan Saloner, James Campbell, Sarah Gollust, Lynn A. Blewett

**Affiliations:** 1 Department of Health Policy and Management, Johns Hopkins Bloomberg School of Public Health, Baltimore, Maryland; 2 Division of Health Policy and Management, University of Minnesota School of Public Health, Minneapolis; 3 State Health Access Data Assistance Center, Division of Health Policy and Management, University of Minnesota School of Public Health, Minneapolis

## Abstract

This article discusses the public’s spending of the stimulus checks issued by the US government during 2020 and 2021.

## Introduction

Material hardships such as food insecurity and problems paying rent have been major concerns during the COVID-19 pandemic.^1,2^ In response, the US Congress authorized substantial direct assistance to households. Stimulus checks were provided in the $2.0 trillion Coronavirus Aid, Relief, and Economic Security Act, passed in March 2020, and the $1.9 trillion American Rescue Plan, passed in March 2021.^3^ Understanding the effect of this relief requires assessing the public’s intentions for these funds and their reported material needs over the first year of the pandemic.

## Methods

We fielded 2 surveys of US adults from April 23 to 27, 2020, and April 15 to 19, 2021,^4^ using the AmeriSpeak Omnibus, a repeated cross-sectional, nationally representative internet/telephone survey. Individuals receiving stimulus checks identified their highest priorities for spending (Table 1). Individuals also reported whether they were confident they could meet the same list of basic material needs over the next month. We compared survey-weighted differences in the outcomes between the 2020 and 2021 groups for all respondents, and also stratified the sample by whether respondents reported their employment was negatively affected by the pandemic (“employment reduction”), a marker of need. We calculated 2-sided *t* tests for differences in proportions, using *P* < .05. Informed consent from participants was obtained by NORC, and the study was approved by the University of Minnesota Institutional Review Board.

**Table 1.** Primary Priority for Spending Stimulus Checks in 2020 and 2021^a^

Characteristic	Employment reduction^b^			No employment reduction			Full sample		
	No. (%)		*P* value^c^	No. (%)		*P* value^c^	No. (%)		*P* value^c^
	2020 (n = 208)	2021 (n = 178)		2020 (n = 621)	2021 (n = 598)		2020 (n = 829)	2021 (n = 776)	
Mortgage or rent	79 (40.0)	39 (18.8)	<.001	103 (18.4)	76 (12.8)	.008	182 (24.3)	115 (14.1)	<.001
Utilities (eg, electricity, water, heat)	30 (18.8)	27 (15.7)	.43	83 (16.7)	62 (11.4)	.008	113 (17.3)	89 (12.4)	.006
Food for myself/family	28 (11.8)	7 (6.1)	.06	75 (14.4)	40 (7.9)	<.001	103 (13.7)	47 (7.5)	<.001
Debt and loans	26 (9.7)	38 (16.7)	.04	134 (19.0)	102 (19.2)	.91	160 (16.4)	140 (18.7)	.24
Medical care or insurance	5 (3.3)	5 (2.5)	.65	26 (3.9)	33 (6.2)	.07	31 (3.7)	38 (5.4)	.11
Savings or investment	24 (9.6)	46 (31.4)	<.001	93 (13.2)	201 (30.1)	<.001	117 (12.2)	247 (30.4)	<.001
Donation	3 (0.9)	0 (0.0)	.20	29 (3.9)	11 (1.2)	.01	32 (3.1)	11 (0.9)	.009
Other	13 (5.8)	16 (8.9)	.35	78 (10.6)	73 (11.2)	.76	91 (9.3)	89 (10.7)	.43

^a^ Priority for spending stimulus checks based on the question in 2021 “the American Rescue Plan Act provides aid to Americans including payment of up to $1,400 per member of a household. What did or do you mostly plan to spend this money on?” and in 2020 “The federal government recently passed a $2 trillion stimulus bill that will provide aid to Americans including payment of up to $1,200 for individuals and $2,400 for married couples. What will be your top priority for how you will spend this money?” Individuals were only included if they stated that they expected to, or already had, received stimulus checks (171 individuals excluded in 2020 and 224 in 2021). All data are survey weighted.

^b^ Employment reduction includes loss of job, reduced hours, and reduced pay owing to the COVID-19 pandemic. This group was 27.4% of the sample in 2020 and 21.8% of the sample in 2021. All other individuals who did not endorse these options were in the group “no employment reduction.”

^c^ *P* values were generated by *t* tests between 2020 vs 2021 values.

## Results

The panel participation rate was 14.4% in 2020 and 20.4% in 2021. The weighted sample in 2020 was 48% female, and 45% were ages 18 to 44 years; characteristics were similar in 2021. In 2020, people with employment reduction reported that the highest priority for their stimulus payment was mortgage/rent (40.0%), followed by utilities (18.8%), food (11.8%), payment of debt/loans (9.7%), savings/investment (8.1%), and medical care (3.3%) (Table 1). In 2021, there was a significant decrease within the employment reduction group for prioritizing paying mortgage/rent (40.0% in 2020 vs 18.8% in 2021, *P* < .001) and also a decrease in prioritizing food, although it did not reach statistical significance (11.8% vs 6.1%, *P* = .06). There were also increases in the proportion reporting that their priority spending was payment of credit card and other debt (9.7% in 2020 vs 16.6% in 2021, *P* = .04) and contributing to savings/investment (8.1% vs 22.9%, *P* < .001). People with no employment reduction in 2021 reported lower priorities compared with 2020 for paying their mortgage or rent, utilities, food, and donations, but higher priorities for savings/investment (see Table 1 for values).

There was a statistically significant decrease among individuals with an employment reduction stating they were “not confident” in their ability to meet 1 or more basic needs, from 54.9% in 2020 to 36.5% in 2021 (*P* < .001) (Table 2). The percent reporting “not confident” in paying for medical care or insurance decreased (33.7% vs 20.2%, *P* = .001). For the full sample, there was improvement in confidence in ability to pay across most categories of expenses including mortgage/rent, utilities, food, and debts/loans. For individuals without an employment reduction, the share reporting “not confident” in 1 or more category of expenses was constant (25.3% in 2020 to 21.5% in 2021; *P* = .12). For those without an employment reduction, the only statistically significant change was in reporting “not confident” in paying for medical care or insurance (13.3% vs 9.0%, *P* = .008).

**Table 2.** Survey Participant Confidence in Ability to Pay for Expenses by Category of Expense in 2020 and 2021^a^

Characteristic^**b**^	Employment reduction^**c**^			No employment reduction			Full sample		
	No. (%)		*P* value^**d**^	No. (%)		*P* value^**d**^	No. (%)		*P* value^**d**^
	2020	2021		2020	2021		2020	2021	
Not confident in >1 options below^e^	100 (54.9)	90 (36.5)	<.001	148 (25.3)	128 (21.5)	.11	248 (33.7)	218 (24.9)	<.001
Mortgage or rent	37 (21.7)	31 (11.4)	.005	43 (8.8)	31 (6.6)	.16	80 (12.7)	62 (7.8)	.001
Utilities (electricity, water, heat, etc)	36 (20.2)	26 (9.7)	.002	31 (5.2)	29 (5.9)	.60	67 (9.3)	55 (6.7)	.04
Food for myself/family	26 (15.9)	20 (6.5)	.002	28 (3.4)	20 (3.3)	.96	54 (6.8)	40 (4.0)	.007
Debt and loans	73 (40.2)	58 (22.3)	<.001	93 (13.9)	87 (14.2)	.88	166 (21.2)	145 (16.0)	.009
Medical care or insurance	62 (33.7)	55 (20.2)	.001	86 (13.3)	77 (9.0)	.008	148 (18.8)	132 (11.5)	<.001

^a^ All data are survey weighted.

^b^ Individuals who said “not applicable” were excluded for each type of expense.

^c^ Employment reduction includes loss of job, reduced hours, and reduced pay owing to the COVID-19 pandemic. All other individuals who did not endorse these options were in the group “no employment reduction.”

^d^ *P* values were generated by *t* tests between 2020 vs 2021 values.

^e^ “Not confident” was defined in response to the question “Over the next 4 weeks, how confident are you that you will be able to pay for the following items?”

## Discussion

A study limitation is the low response rate, an issue that has generally affected surveys during the COVID-19 pandemic.^5^ The significant decrease in material hardship between April 2020 and April 2021 reflects an improving economic situation for US households, and may explain why more households in 2021 intended to use their stimulus checks for savings over basic needs. Improvements were especially pronounced for those experiencing COVID-19 employment reduction, but substantial unmet need remained in 2021 for this group. A slow economic recovery may prolong financial insecurity for this group.^1^
